# Imaging in otosclerosis: A pictorial review

**DOI:** 10.1007/s13244-014-0313-9

**Published:** 2014-02-09

**Authors:** Bela Purohit, Robert Hermans, Katya Op de beeck

**Affiliations:** Department of Radiology, University Hospitals Leuven, 3000 Leuven, Belgium

**Keywords:** Otosclerosis, Fenestral, Retrofenestral, HRCT temporal bone, Stapedectomy

## Abstract

Otosclerosis is an otodystrophy of the otic capsule and is a cause of conductive, mixed or sensorineural hearing loss in the 2nd to 4th decades of life. Otosclerosis is categorised into two types, fenestral and retrofenestral. Imaging plays an important role in the diagnosis and management of otosclerosis. High-resolution CT (HRCT) of the temporal bone using 1-mm (or less) thick sections is the modality of choice for assessment of the labyrinthine windows and cochlear capsules. MRI has limited application in the evaluation of the labyrinthine capsules but is useful for assessment of the cochlear lumen prior to cochlear implantation in patients with profound hearing loss. The treatment of fenestral otosclerosis is primarily surgical with stapedectomy and prosthesis insertion. Patients with retrofenestral otosclerosis and profound hearing loss are treated medically using fluorides, but may derive significant benefit from cochlear implantation. This pictorial review aims to acquaint the reader with the pathology and clinical features of otosclerosis, the classical imaging appearances on CT and MRI, a radiological checklist for preoperative CT evaluation of otosclerosis, imaging mimics and a few examples of post-stapedectomy imaging and complications.

*Teaching points*

• *Otosclerosis causes conductive, sensorineural and mixed hearing loss in adults*.

• *HRCT of the temporal bone is the diagnostic imaging modality of choice*.

• *Stapedectomy is used to treat fenestral otosclerosis*.

• *Fluorides and cochlear implantation are used to treat retrofenestral otosclerosis*.

## Introduction

Otosclerosis is a unique autosomal dominant otodystrophy of the otic capsule. It is also called ‘otospongiosis’ as it is characterised by replacement of the normal ivory-like enchondral bone by spongy vascular bone. The decalcified foci tend to recalcify, becoming less vascular and more solid. Patients typically present in the 2nd- 4th decades of life with conductive hearing loss (CHL), sensorineural hearing loss (SNHL) or mixed hearing loss (MHL) and/or tinnitus. Otosclerosis is commoner in Caucasians as compared to blacks, Native Americans and Asians. The disease is more common in women and commonly bilateral (85 %). Otosclerosis is categorised into two types, fenestral and retrofenestral/cochlear. Retrofenestral otosclerosis rarely occurs without fenestral involvement; hence these manifestations are considered to be a continuum rather than two separate entities [1–4].

## Fenestral Otosclerosis

### Pathology, clinical findings and imaging

**Fig. 1:**
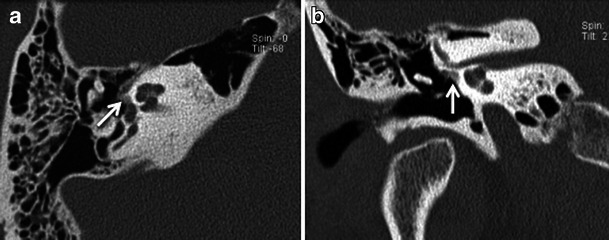
Axial (**a**) and coronal (**b**) HRCT images of the right temporal bone in an adult patient with right-sided CHL. A hypodense demineralised plaque (*arrow*) is noted in the region of the fissula ante fenestram in keeping with fenestral otosclerosis

**Fig. 2:**
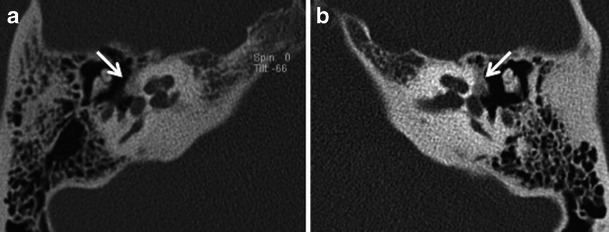
Axial HRCT images of the *right* (**a**) and *left* (**b**) temporal bone in an adult patient with bilateral CHL. Hypodense demineralised plaques (*arrows*) are noted in bilateral fissula ante fenestram regions in keeping with bilateral fenestral otosclerosis

**Fig. 3:**
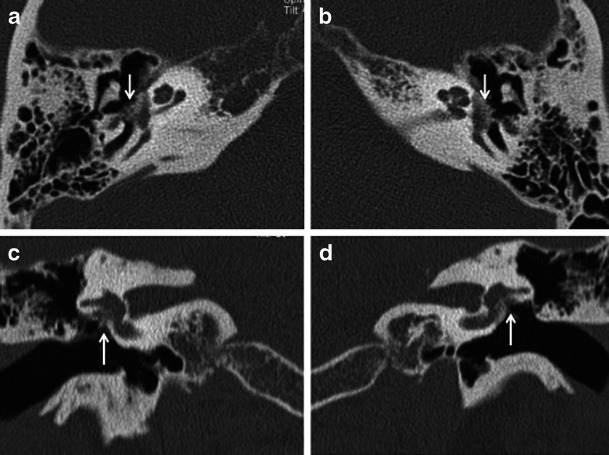
Axial (**a**,**b**) and coronal (**c**,**d**) HRCT images of the *right* and *left* temporal bone in an adult patient with bilateral severe CHL. Heaped-up bony otosclerotic plaques are noted causing severe bilateral oval window narrowing (*arrows*)

The more common fenestral type of otosclerosis involves the lateral wall of the bony labyrinth. Histologically, demineralised foci of spongy new bone typically occur in the region of the embryonic fissula ante fenestram, which is a cleft of fibrocartilagenous tissue between the inner and middle ear, just anterior to the oval window (Fig. 1). Bilateral involvement is common (Fig. 2). The promontory, round window niche and tympanic segment of the facial nerve canal can also be involved [1–3]. The disease gradually extends to involve the entire footplate of the stapes and may subsequently involve the cochlea. Heaped-up bony plaques formed in the healing phase typically cause narrowing of the oval and round windows. Involvement of the annular ligament leads to mechanical fixation of the stapedo-vestibular joint, which is responsible for the typical CHL/audiometric air-bone gap (Carhart’s notch) [1–5]. Complete obliteration of the oval window may occur in 2 % cases (Fig. 3). This rarely is associated with secondary torsional subluxation of the incus [2]. Otosclerosis can sometimes present as isolated round window involvement without pericochlear or oval window involvement [6].

The classical clinical findings include progressive CHL up to about 50–60 dB, absent stapedial reflexes, a normal tympanic membrane and no evidence of middle ear inflammation [1–5].

Imaging is usually not pursued in patients with uncomplicated CHL and characteristic clinical findings. The treatment of fenestral otosclerosis is primarily surgical with stapedectomy and stapes prosthesis insertion [1–5].

High-resolution CT (HRCT) of the temporal bone is the modality of choice for the preoperative evaluation of otosclerosis. Typically, very thin axial sections are obtained on a multidetector CT scanner, followed by axial and coronal reformats, respectively in the plane of and perpendicular to the lateral semicircular canal. If needed, additional reformats can be made, for example along the plane of the stapedial suprastructure. All studies are performed without contrast and the entire petrous temporal bone is included in the sections. Demineralised hypodense fenestral otosclerotic foci are best seen on axial HRCT because of the anteroposterior orientation of the oval window and stapes crura. Fenestral otosclerotic foci as small as 1 mm in size can be diagnosed on HRCT [1–5]. Some authors have mentioned a correlation between the size of the fenestral otosclerotic focus and the air-bone gap [5].

**Fig. 4:**
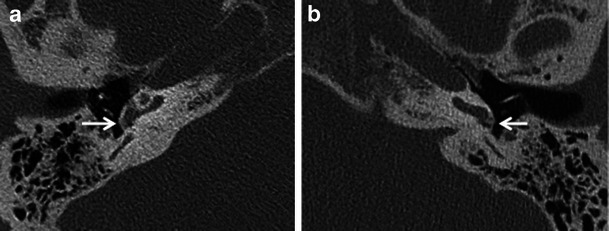
Axial HRCT images of the *right* (**a**) and *left* (**b**) temporal bone in a patient with bilateral fenestral otosclerosis. Otosclerotic plaques are noted causing bilateral round window narrowing (*arrows*), *right* more than *left*

**Fig. 5:**
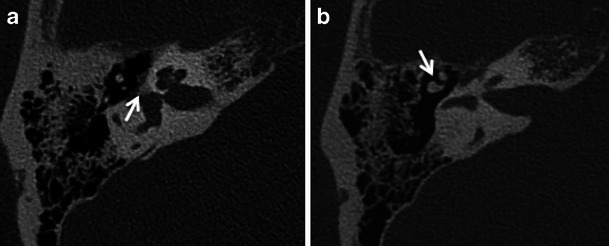
**a** Axial HRCT image of the right temporal bone in an adult patient with progressive right-sided CHL and remote history of ipsilateral head injury. A hypodense demineralised otosclerotic plaque (*arrow*) is noted in the fissula ante fenestram. **b** Axial HRCT image of the same patient as (**a**) at a slightly higher level. There is also evidence of malleo-incudal dislocation (*arrow*)

**Fig. 6:**
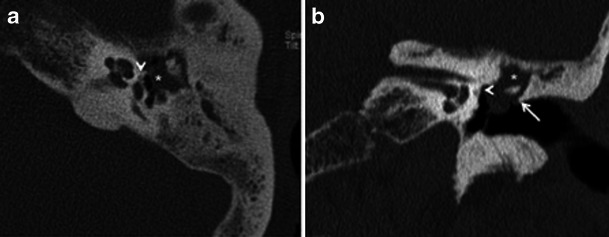
Axial (**a**) and coronal (**b**) HRCT images of the left temporal bone in an adult patient with left-sided CHL and previous history of left-sided otitis media. The soft-tissue density noted in the attic (*asterisk*) causing blunting of the scutum (*arrow*) is in favour of a cholesteatoma. In addition, a tiny hypodense fenestral otosclerotic focus (*arrowheads*) is noted anterior to the oval window

Apart from assessing the size and location of plaques and the narrowing of the oval window, the radiologist must evaluate the status of the round window, facial nerve canal, jugular bulb, middle ear cavity, ossicular chain and inner ear. Obliteration of the round window by the otosclerotic process may reduce the efficacy of stapedectomy and must be mentioned in the report (Fig. 4) [1–3]. Other or associated causes of CHL and SNHL must be ruled out prior to surgery. These include congenital ossicular fusion, ossicular discontinuity (Fig. 5), inflammatory middle ear disease (Fig. 6) and inner ear pathology such as acoustic neuroma and labyrinthitis ossificans [2]. Table 1 describes a recommended checklist for reporting preoperative HRCT of the temporal bone with clinical and surgical relevance of each of the points.

**Table 1:** Reporting checklist for preoperative HRCT of the temporal bone in otosclerosis

	Reporting points	Clinical and surgical relevance
1.	Size and location of plaques	Size and location of plaques may correlate with severity of CHL/air-bone gap
2.	Status of oval window	Complete obliteration may require surgical drilling prior to prosthesis insertion
3.	Status of round window	Obliteration may result in a poor result after stapedectomy
4.	Facial nerve canal	Floppy facial nerve may complicate oval window surgery or render this impossible
5.	Concurrent middle ear pathology	Inflammatory disease must be treated prior to surgery
6.	Ossicular chain integrity	Ossicular fixation, fusion and fracture may compound CHL
7.	Sinus plate and jugular bulb	Dehiscent jugular bulb may complicate surgery
8.	Inner ear pathology	Congenital cochlear and inner ear anomalies may preclude surgery
9.	Opposite ear	Disease is bilateral in 80-85 % cases, even in absence of symptoms

**Fig. 7:**
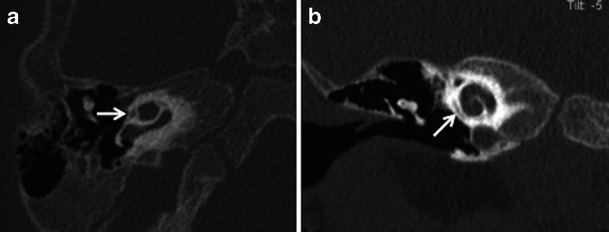
Axial (**a**) and coronal (**b**) HRCT images of the right temporal bone in a child with suspected left SNHL. The small lucency seen around the cochlea (*arrow*) on both the axial and coronal images is in keeping with a cochlear cleft (normal variant)

False-negative CT findings may occur in some cases of fenestral otosclerosis in the sclerotic phase when there are no irregularities of the bone contour [5]. The imaging differentials of fenestral otosclerosis are few. The cochlear cleft is a small non-osseous space in the otic capsule in the region of the fissula ante fenestram. It is a normal variant, commonly seen in children, and its incidence decreases with age. An inexperienced reader may mistake a cochlear cleft for a demineralised focus in the region of the fissula ante fenestram (Fig. 7) [2, 3, 7]. Tympanosclerois with post-inflammatory fixation of the stapes footplate may present clinically with identical CHL, especially with a healed tympanic membrane. This may cause a diagnostic dilemma in certain cases, although, this can be differentiated on CT by observing signs of inflammation in the middle ear and an underpneumatised mastoid [2, 3].

### Post stapedectomy imaging and complications

**Fig. 8:**
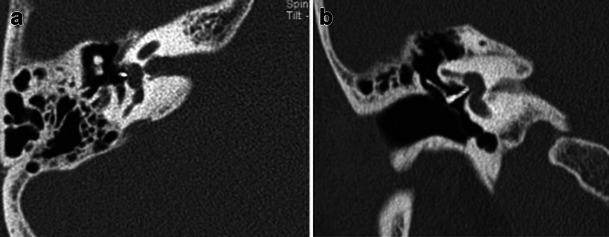
Axial (**a**) and coronal (**b**) HRCT images of the right temporal bone in a patient with stapedectomy. The radiodense stapes prosthesis is well positioned with its distal end against the oval window

**Fig. 9:**
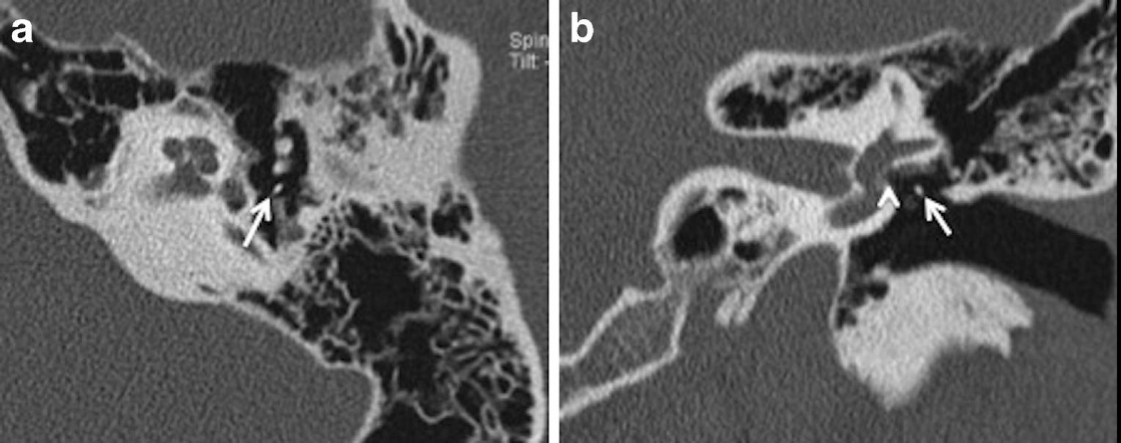
Axial (**a**) and coronal (**b**) HRCT images of the left temporal bone in a patient with persistent CHL, post stapedectomy. The stapes prosthesis (*arrow*) is dislocated posteriorly in relation to the oval window (*arrowhead*) with complete loss of contact

**Fig. 10:**
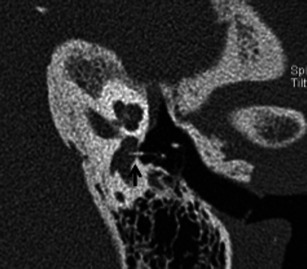
Para-axial HRCT image of the left temporal bone in a patient with severe vertigo, post stapedectomy. The stapes prosthesis is dislocated and lies partly within the vestibule (*arrow*)

**Fig. 11:**
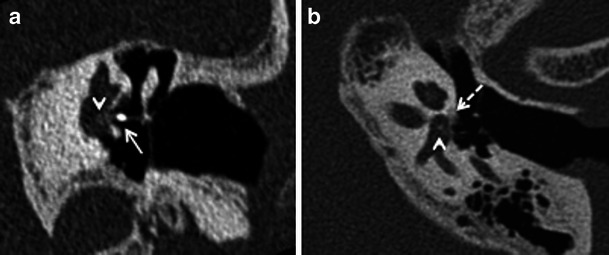
Para-coronal (**a**) and para-axial (**b**) HRCT images of the left temporal bone in a patient with persistent vertigo and left-sided SNHL after left stapedectomy. The stapes prosthesis (*arrow*) is well positioned. However a small sclerotic focus is seen within the vestibule (*arrowheads*) in keeping with labyrinthitis ossificans. A small fenestral otosclerotic focus (*dashed arrow*) is seen in the para-axial view

Stapedectomy is commonly combined with insertion of a stapes prosthesis in order to restore ossicular chain continuity. The use of a radiodense stapes prosthesis helps radiological evaluation on HRCT (Fig. 8). Stapedectomy with prosthesis insertion is associated with some inherent complications. The common causes for recurrent CHL, vertigo and SNHL after stapes surgery include complete displacement of the prosthesis (Fig. 9), prosthesis displacement into the vestibule (Fig. 10), perilymphatic fistula, and development of reparative granulomas and labyrinthitis (Fig. 11) HRCT helps to evaluate the position of the prosthesis and rule out common complications. Additional MRI may act as an adjunct to rule out labyrinthitis ossicificans. MRI can rule out fibrotic changes in the labyrinth while ossifications are diagnosed exclusively by CT [2, 3, 8–10]. Repeat surgery may be mandatory for treatment of a dislocated prosthesis or closure of a perilymph fistula [8–10].

## Retrofenestral or cochlear otosclerosis

### Pathology, clinical findings and imaging

Retrofenestral or cochlear otosclerosis is much less common; however, it is nearly always associated with fenestral otosclerosis. Patients typically present with bilaterally symmetrical SNHL or MHL. Pulsatile tinnitus is also known to occur. Cochlear otosclerosis represents a continuum of the fenestral otosclerotic process. Histologically, foci of demineralised spongy vascular bone are seen in the cochlear capsule, which may extend around the vestibule, semicircular canals and internal auditory canal. The promontory may show a pink hue when seen through the tympanic membrane, called the Schwartze sign. Direct injury to the cochlea and spiral ligament due to the lytic process or release of proteolytic enzymes is implicated as a possible cause for the SNHL [1–4].

**Fig. 12:**
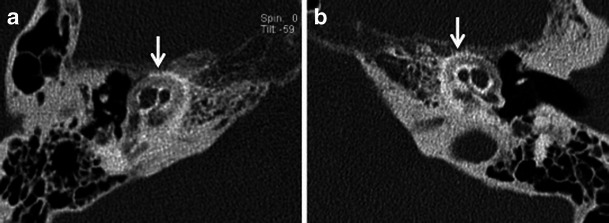
Axial HRCT images of the *right* (**a**) and *left* (**b**) temporal bone in an adult patient with severe bilateral SNHL. The bilateral pericochlear hypodense ‘double ring’ (*arrow*) is in keeping with bilateral cochlear otosclerosis

**Fig. 13:**
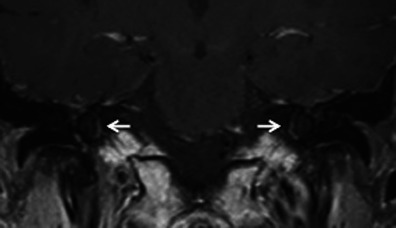
Coronal contrast-enhanced MR image in a patient with left-sided SNHL. Bilateral pericochlear ring-like enhancement (*arrow*) is suggestive of bilateral cochlear otosclerosis, which was further proven by HRCT

**Fig. 14:**
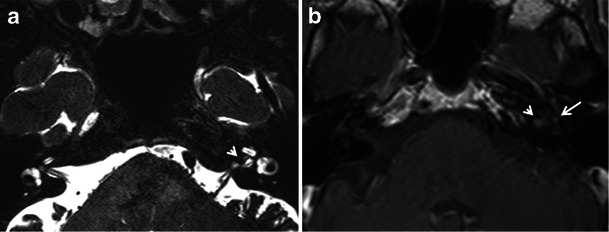
**a** Axial CISS MR image of the skull base in an adult patient with left-sided SNHL. A small hypointense filling defect is seen in the left internal auditory canal (*arrowhead*), which may be suggestive of an acoustic neuroma. **b** Axial contrast-enhanced MR image of the same patient as (**a**), at the same level. The previously noted filling defect in the left internal auditory canal shows post-contrast enhancement, which indicates a small acoustic neuroma (*arrowhead*). There is also a suggestion of enhancement in the left pericochlear region and in the region of the left fissula ante fenestram (*arrow*), which suggests associated otosclerosis is present

**Fig. 15:**
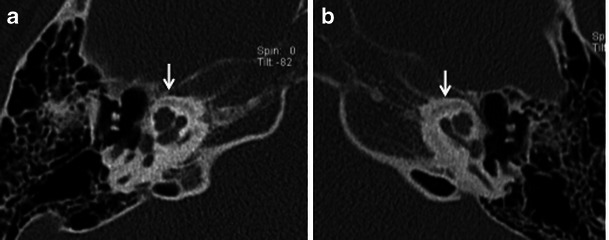
Axial HRCT images of the *right* (**a**) and *left* (**b**) temporal bone in a young adult with known osteogenesis imperfecta tarda and bilateral SNHL. Bilateral pericochlear ring-like hypodensity (*arrows*) closely mimics cochlear otosclerosis

**Fig. 16:**
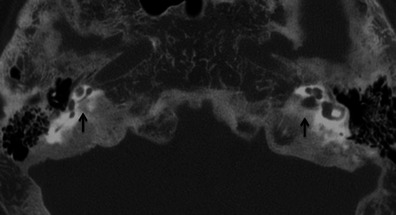
Axial HRCT of the skull base in an adult patient with known Paget’s disease. The hypodense appearance of bilateral otic capsules (*arrows*) may mimic otosclerosis; however the diffuse skull base involvement indicates the true pathology

HRCT adequately demonstrates the demineralised foci in the otic capsule. The classical imaging appearance of cochlear otosclerosis on HRCT is a distinctive pericochlear hypodense double ring (which is also known as the 4th ring of Valvassori) (Fig. 12). Bilateral symmetry is common [1–4]. A CT grading of otosclerosis has been proposed by Symons/Fanning and is described in Table 2 [4]. Some authors mention that the severity of cochlear disease correlates with early onset as well as increasing severity of SNHL [4]. At times, MRI is performed prior to CT for assessing the cause of SNHL. A ring of pericochlear and perilabyrinthine intermediate signal on T1-weighted images and mild-moderate post-gadolinium enhancement has been reported in cochlear otosclerosis, more so in the active phase (Fig. 13) [2, 3, 11, 12]. MRI may also pick up other unassociated inner ear pathologies (Fig. 14). The HRCT appearance of cochlear otosclerosis is rarely mimicked by various diseases that demineralise the otic capsule, including osteogenesis imperfecta (Fig. 15) and Paget’s disease (Fig. 16). However the clinical manifestations and involvement of other bones suffice to differentiate these pathological conditions from cochlear otosclerosis [2–4, 11, 13].

**Table 2:** CT grading of otosclerosis (Symons/Fanning 2005)

CT grading of otosclerosis	Location of plaques
Grade 1	Solely fenestral
Grade 2	Patchy localised cochlear disease (+/− fenestral involvement) • To basal turn (grade 2A) • To middle turn (grade 2B) • Around lateral aspect of basal, middle, apical turns (grade 2C)
Grade 3	Diffuse confluent cochlear involvement (+/− fenestral involvement)

**Fig. 17:**
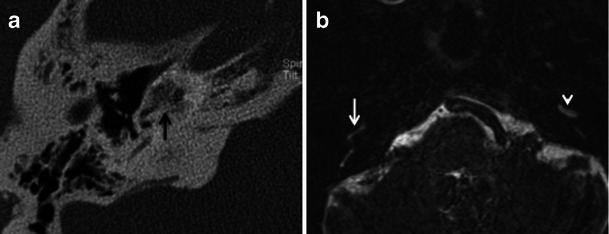
**a** Axial HRCT of the right temporal bone in a patient with known bilateral cochlear otosclerosis. There is almost complete obliteration of the basal turn of the right cochlea (*arrow*) by the otosclerotic process. **b** Axial MRI of the temporal bones in the same patient as (17a) at the level of the cochlea. There is significant obliteration of the fluid space in the basal turn of the right cochlea (*arrow*). The normal fluid space in the basal turn of the left cochlea (*arrowhead*) is shown for comparison

New bone formation (membranous labyrinth ossification) is unusual with cochlear otosclerosis and is invariably limited to the basal turn of the cochlea [2, 14, 15]. This may be a relative contraindication for cochlear implantation (CI). In this setting, MRI is useful for assessment of the cochlear lumen (Fig. 17) [2, 3, 14, 15].

### Treatment

Patients with cochlear otosclerosis are usually treated medically using fluorides [2, 3, 5, 11, 16]. Fluoride therapy may limit the growth of active otosclerotic foci and thereby prevent progression of SNHL [5]. However patients with bilateral profound SNHL may derive significant benefit from CI [2–4, 14, 15]. CI surgery in patients with otosclerosis may be challenging. There is high risk of partial insertion and misplacement of electrode arrays requiring revision surgery. This is ascribed to the ossification of the scala tympani in the basal turn of the cochlea [14, 15].

## Conclusion

This article aims to serve as a concise review of the pathology and common imaging appearances of otosclerosis, imaging mimics and certain uncommon postoperative appearances and complications associated with otosclerosis.

## Abbreviations

HRCT: high-resolution CT
CHL: conductive hearing loss
SNHL: sensorineural hearing loss
MHL: mixed hearing loss
CI: cochlear implantation
